# Epidemiology of invasive group B streptococcal disease in infants from urban area of South China, 2011–2014

**DOI:** 10.1186/s12879-017-2811-0

**Published:** 2018-01-08

**Authors:** Xiaoshan Guan, Xiaoping Mu, Wenjing Ji, Chunlei Yuan, Ping He, Lian Zhang, Yanfen Huang, Juan Li, Jianfeng Chen, Huamin Zhong, Shuyin Pang, Nan Tan, Qiulian Deng, Kankan Gao, Yu-Ping Huang, Chien-Yi Chang, Haiying Liu

**Affiliations:** 1 Clinical Laboratory, Guangzhou Women and Children’s Medical Center, Guangzhou Medical University, No.9 Jinsui Road, Guangzhou, Guangdong Province 510623 People’s Republic of China; 2 Clinical Laboratory, Guangdong Women and Children’s Hospital, Guangzhou, Guangdong China; 3 Department of Pharmacy Administration and Clinical Pharmacy, School of Pharmacy, Xi’an Jiaotong University, Xi’an, Shaanxi China; 4 The Center for Drug Safety and Policy Research, Xi’an Jiaotong University, Xi’an, Shaanxi China; 5 The Global Health Institute, Xi’an Jiaotong University, Xi’an, Shaanxi China; 6 Clinical Laboratory, Zhongshan Boai Hospital, No.6 Chenggui Road, Zhongshan, Guangdong Province 528403 People’s Republic of China; 7 Department of Gynecology and Obstetrics, Guangzhou Women and Children’s Medical Center, Guangzhou Medical University, Guangzhou, Guangdong China; 8 Department of Neonatalogy, Guangzhou Women and Children’s Medical Center, Guangzhou Medical University, Guangzhou, Guangdong China; 9 Guangzhou Institute of Pediatrics, Guangzhou Women and Children’s Medical Center, Guangzhou Medical University, Guangzhou, Guangdong China

**Keywords:** Infants, Group B *Streptococcus*, Neonatal infections, Serotype, Multilocus sequence type (MLST), Antimicrobial resistance

## Abstract

**Background:**

Group B *Streptococcus* (GBS) is a leading cause of morbidity and mortality in infants in both developed and developing countries. To our knowledge, only a few studies have been reported the clinical features, treatment and outcomes of the GBS disease in China. The severity of neonatal GBS disease in China remains unclear. Population-based surveillance in China is therefore required.

**Methods:**

We retrospectively collected data of <3 months old infants with culture-positive GBS in sterile samples from three large urban tertiary hospitals in South China from Jan 2011 to Dec 2014. The GBS isolates and their antibiotic susceptibility were routinely identified in clinical laboratories in participating hospitals. Serotyping and multi-locus sequence typing (MLST) were also conducted for further analysis of the neonatal GBS disease.

**Results:**

Total 70 cases of culture-confirmed invasive GBS infection were identified from 127,206 live births born in studying hospitals, giving an overall incidence of 0.55 per 1000 live births (95% confidence interval [CI] 0.44–0.69). They consisted of 49 with early-onset disease (EOD, 0.39 per 1000 live births (95% CI 0.29–0.51)) and 21 with late-onset disease (LOD, 0.17 per 1000 live births (95% CI 0.11–0.25)). The incidence of EOD increased significantly over the studying period. Five infants (4 EOD and 1 LOD) died before discharge giving a mortality rate of 7.1% and five infants (7.1%, 2 EOD and 3 LOD) had neurological sequelae. Within 68 GBS isolates from GBS cases who born in the studying hospitals or elsewhere, serotype III accounted for 77.9%, followed by Ib (14.7%), V (4.4%), and Ia (2.9%). MLST analysis revealed the presence of 13 different sequence types among the 68 GBS isolates and ST-17 was the most frequent sequence type (63.2%). All isolates were susceptible to penicillin, ceftriaxone, vancomycin and linezolid, while 57.4% and 51.5% were resistant to erythromycin and clindamycin, respectively.

**Conclusions:**

This study gains the insight into the spectrum of GBS infection in south China which will facilitate the development of the guidance for reasonable antibiotics usage and will provide evidence for the implementation of potential GBS vaccines in the future.

**Electronic supplementary material:**

The online version of this article (10.1186/s12879-017-2811-0) contains supplementary material, which is available to authorized users.

## Background

Group B *Streptococcus* (GBS) is recognized as a leading cause of neonatal morbidity and mortality around the world [1–4]. The incidence varies significantly by region. A systematic review and meta-analysis reported an estimated global incidence of GBS in infants <3 months old of 0.53 per 1000 live births (95% confidence interval [CI] 0.44–0.62) [5]. The incidence was highest in Africa, with 1.21 per 1000 live births, followed by the Americas (0.67 per 1000 live births) and Europe (0.57 per 1000 live births) [5]. Southeast Asia had the lowest incidence, at 0.02 per 1000 live births [5]. However, data from Asia are very limited and the disease burden in Mainland China remains unclear [5, 6].

The Center for Disease Control and Prevention (CDC) in US has issued a perinatal disease prevention strategy in 1996 with two revisions in 2002 and 2010 suggesting universal culture-based screening of pregnant women and intrapartum antibiotic prophylaxis (IAP) for GBS colonized women as an approach for neonatal GBS prevention. The implementation of the guidelines decreased the incidence of early-onset GBS disease (EOD; occurring in infants <7 days old) from 1.7 per 1000 live births in 1993 to 0.25 per 1000 live births by 2014 [7–9]. Specific guidelines such as IAP for GBS prevention are lacking in China and only few hospitals strictly follow the guideline issued by the US CDC. The three study hospitals did not implement IAP during the studying period.

Our previous study, the first prospective observational study conducted in Mainland China, showed an incidence of GBS of 0.28 per 1000 live births (95% CI 0.08–1.03, 2/7061 subjects) [10]. However, this 6-month study included only two urban hospitals [10]. To obtain more precise incidences of invasive GBS in Guangdong province, we retrospectively collected data of cases with culture-confirmed invasive GBS disease among <3 months old infants from three large urban tertiary hospitals for 2011–2014. This study was to investigate the epidemiology of GBS infections in south China and to provide evidence for developing preventive interventions and the introduction of GBS vaccines in the future.

## Methods

### Study design

This retrospective study was conducted within three large urban tertiary hospitals in Guangdong (Guangzhou Women & Children’s Medical Center, Guangdong Provincial Maternity & Children’s Hospital, and Zhongshan Boai Hospital) from Jan 2011 to Dec 2014. Skilled attendants in these hospitals provide maternity services to pregnancies, intensive care units in these hospitals also provide neonatal and pediatric medical services. This study was approved by the local ethical committees of three study hospitals.

### Data collection

GBS cases were identified from the microbiology laboratory databases of the studying hospitals. All of the study hospitals used automated enrichment culture methods to identify GBS. A GBS case was defined as a < 3 months old infant with a confirmed positive GBS culture from a sterile sample accompanied any of clinical signs or symptoms of sepsis, meningitis or pneumonia. EOD was defined as isolation of GBS from the infants within the first 6 days after birth, and late-onset disease (LOD) was defined as isolation of GBS from the infants 7–89 days after birth. Only culture-confirmed GBS cases born in the studying hospitals were included for the estimation of incidence and evaluation of clinical outcomes at discharge e.g. survived, survived with sequelae and died. The medical records of GBS cases were reviewed and extracted. The numbers of live births per year between 2011 and 2014 were provided by the statistical departments of studying hospitals.

### GBS strain analysis

For suspected cases, blood and/or cerebrospinal fluid (CSF) samples were taken into culture bottles and sent to laboratories according to operation manuals of blood culture system (BD Bactec™ 9210 or BioMerieux Bact/ALERT). The *Streptococcus* genus was identified using Gram stain, colony morphology, haemolysis, and a catalase test. API 20STREP, an automated identification panel for Gram-positive bacteria, was then employed (VITEK 2 COMPACT, BioMerieux, France). GBS isolates from culture-confirmed cases born elsewhere but transferred to the studying hospitals for medical treatment after delivery also were enrolled into strain analysis. Antimicrobial susceptibility to penicillin, ceftriaxone, vancomycin, linezolid, ofloxacin, sulfamethoxazole, tetracycline, erythromycin, clindamycin, was examined using the AST-GP67 (VITEK 2 COMPACT) or manual Kirby-Bauer disc diffusion, as per interpretive standards established by the Clinical and Laboratory Standards Institute (US) [11]. GBS isolates were recovered in trypticase soy agar supplemented with 5% sheep blood. Lancefield serotyping was performed using a Strep-B-Latex® rapid latex agglutination test kit (Statens Serum Institute, Hillerød, Denmark) according to the manufacturer’s instructions. Multi-locus sequence typing (MLST) was performed by sequencing the internal fragments of seven house-keeping genes (*adhP*, *pheS*, *atr*, *glnA*, *sdhA*, *glcK* and *tkt*). PCR products were purified and sequenced in both directions by BGI Tech Solutions Co.,Ltd.. Alleles and sequence types (STs) were determined using the *S. agalactiae* MLST website (http://pubmlst.org/sagalactiae/).

### Statistical analysis

The incidence per 1000 live births was calculated by dividing the total number of confirmed GBS cases born in the studying hospitals by the total number of infants born in the studying hospitals and multiplying by 1000. For both the incidence and mortality, 95% confidence intervals (CI) were calculated using the Wilson interval method. The percentage changes were used to compare the incidences in each calendar year during the period of this study. The mortality rate was defined as the percentage (%) of the GBS cases born in studying hospitals, in which the infants died before discharge. The collected strains isolated from GBS cases born in the studying hospitals or elsewhere were enrolled for further analyses of serotyping, MLST and antimicrobial susceptibility. Serotype distribution is described as a frequency distribution (number and percentage). All analyses were completed with SPSS 19.0 (IBM Corp., Armonk, NY, US), and *P* < 0.05 was considered statistically significant.

## Results

### Incidence

**Fig. 1:**
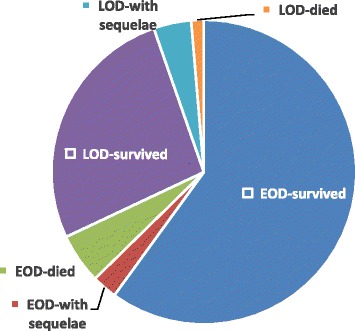
Clinical outcomes of culture-confirmed GBS cases identified from infants <3 months born in the three studying hospitals. This figure indicates number of GBS cases was identified from 127,206 live births, mortality rate of EOD and LOD, and proportion of cases with neurological sequelae, respectively. EOD, early-onset disease; LOD, late-onset disease

Of the 127,206 live births in the three studying hospitals between 2011 and 2014, 70 GBS cases were identified, 49 (70.0%) were EOD, and 21 (30.0%) were LOD (Table 1). Among 70 GBS cases, 11 (15.7%) were born less than 37 weeks gestational age and 9 (12.9%) weighed less than 2500 g at delivery. The rates of preterm or low birth weight in the EOD cases didn’t differ from that of the LOD (*P* > 0.05). Of the total 70 cases, 5 (4 EOD and 1 LOD) died before discharge, for a mortality rate of 7.1% (95% CI 1.1–13.2), and 5 (7.1%, 2 EOD and 3 LOD) had neurological sequelae (Fig. 1, Table 1).

**Table 1:** Demographics of infants with invasive GBS infection in urban area of South China, 2011–2014

	Early-onset Disease (*n* = 49)	Late-onset Disease (*n* = 21)	Total (*n* = 70)
Sex (female/male)	16/33	12/9	28/42
Preterm (gestational age < 37 weeks)	7 (14.3%)	4 (19.0%)	11 (15.7%)
Low birth weight (<2500 g)	5 (10.2%)	4 (19.0%)	9 (12.9%)
Bacterial isolation site			
Blood alone	46 (93.9%)	16 (76.2%)	62 (88.6%)
CSF+blood^a^	2 (4.1%)	0	2 (2.9%)
CSF alone	1 (2.0%)	5 (23.8%)^b^	6 (8.6%)
Incidence (per 1000 live births,95%CI)	0.39 (0.29–0.51)	0.16 (0.11–0.25)	0.55 (0.44–0.69)
Clinical outcomes			
Died	4 (8.2%)	1 (4.8%)	5 (7.1%)
Survived with sequelae	2 (4.1%)	3 (14.3%)	5 (7.1%)

^a^*CSF* cerebrospinal fluid;

^b^Compared with early-onset disease, Fisher exact test, X^2^ = 6.3281, *P* = 0.0119

**Fig. 2:**
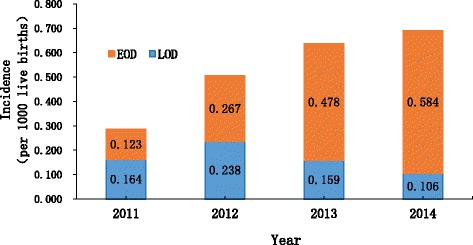
Incidence of invasive group B streptococcal disease in infants younger than three months in South China, 2011–2014. This figure describes the overall incidence of invasive GBS infection during 2011–2014, and incidence for EOD and LOD, respectively. It shows the increase for EOD from 2011 to 2014 was significant, while the incidence of LOD was more stable

The overall incidence of invasive GBS infection was 0.55 per 1000 live births (95% CI 0.44–0.69) during 2011–2014. The disease incidences increased significantly from 0.29 per 1000 live births (95% CI 0.14–0.59) in 2011 to 0.69 per 1000 live births (95% CI 0.48–1.01) in 2014 (*P* < 0.05) (Fig. 2). Incidences for EOD and LOD were 0.39 per 1000 live births (95% CI 0.29–0.51) and 0.17 per 1000 live births (95% CI 0.11–0.25), respectively. The incidences of EOD in 2011, 2012, 2013, and 2014 were 0.12, 0.27, 0.48, and 0.58 per 1000 live births, respectively (Fig. 2). The increase for EOD from 2011 to 2014 was significant (χ^2^ for trend, 10.158; *P* = 0.017). While the incidence of LOD was more stable and ranged from 0.11 to 0.24 per 1000 live births during the studying period.

### Distribution of serotypes and MLSTs

**Fig. 3:**
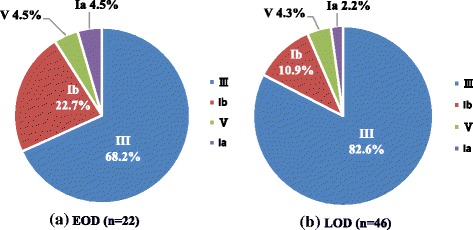
Distribution of group B streptococcus serotypes in infants younger than three months with invasive infection. This figure shows GBS serotype distribution among study infants. Serotype III was the most commonly identified serotype, followed by Ib, V, and Ia

Within the retrospective study time, a total of 68 isolates had been collected and stored from GBS infected cases from infants who were born in the studying hospitals or elsewhere but transferred to the studying hospitals for medical treatment after delivery. To further characterise the spectrum of GBS disease, the 68 GBS isolates were enrolled for strain analyses (Table 2). Four serotypes were identified, and all of the isolates were determined. Serotype III (53, 77.9%) was the most commonly identified serotype among the GBS isolates, followed by Ib (10, 14.7%), V (3, 4.4%), and Ia (2, 2.9%). Serotype III was present in 68.2% of the 22 EOD cases and 82.6% of the 46 LOD cases (χ^2^ = 0.303, *P* > 0.05, Fig. 3). MLST analysis revealed the presence of 13 different STs among the 68 GBS isolates (Table 3). The most frequent type was ST-17(43, 63.2%), followed by ST-12(5, 17.6%), ST-19(4, 5.9%), ST-23(3, 4.4%) and ST-1(3, 4.4%). All the ST-17 were identified in serotype III isolates, which accounted for 81.1% of ST of serotype III.

**Table 2:** Serotyping analysis of invasive GBS infection in urban area of South China, 2011–2014

	Early-onset Disease (*n* = 22)	Late-onset Disease (*n* = 46)	Total^a^(*n* = 68)
Sex (female/male)	6/16	25/21	31/37
Bacterial isolation site			
Blood	20 (90.9%)	28 (60.9%)	48 (70.6%)
CSF^b^	2 (9.1%)	18 (39.1%)	20 (29.4%)
Serotype^c^			
Ia	1 (4.5%)	1 (2.2%)	2 (2.9%)
Ib	5 (22.7)	5 (10.9%)	10 (14.7%)
III	15 (68.2%)	38 (82.6%)	53 (77.9%)
V	1 (4.5%)	2 (4.3%)	3 (4.4%)

^a^The analyzed 68 GBS isolates were collected during study period, including from cases born in the studying hospitals or born elsewhere but transferred to the studying hospitals for medical treatment after delivery

^b^*CSF* cerebrospinal fluid

^c^Compared with early-onset disease, serotypes distribution for late-onset disease was not statistically different (X^2^ = 2.1062, *P* = 0.5506)

**Table 3:** Multi-locus sequence typing of invasive GBS infection in urban area of South China, 2011–2014

Serotype	Total No	Sequence Type	Isolates number
III	53	ST-17	43
		ST-19	4
		ST-399	2
		ST-27	1
		ST-55	1
		ST-562	1
		ST-651	1
Ib	10	ST-12	5
		ST-10	1
		ST-23	1
		ST-357	2
		ST-579	1
V	3	ST-1	3
Ia	2	ST-23	2

### Antibiotic susceptibility

In prevention of perinatal group B streptococcal disease, penicillin, ampicillin and cefazolin are recommended for IAP or management of newborns with respect to risk for early-onset GBS disease; second-line drugs such as vancomycin, macrolides (i.e., erythromycin, azithromycin, and clarithromycin), and lincosamides (clindamycin and lincomycin) may be used as alternative in a patient allergic to penicillin or cephalosporins. Penicillin G is the drug of choice for treatment of GBS infections. As shown in Table 4, all the 68 GBS isolates were susceptible to penicillin, ceftriaxone, vancomycin, and linezolid. There were 4 (5.9%) and 2 (2.9%) isolates were resistant to ofloxacin and sulfamethoxazole, respectively. The rates of resistance to erythromycin and clindamycin were 57.4% (39/68) and 51.5% (35/68), respectively. The isolates showed a high resistance (65/68, 95.6%) to tetracycline. 33.8% (23/68) isolates showed multi-drug resistance to tetracycline, lincosamides and macrolides. However, difference in resistance to erythromycin and clindamycin couldn’t be observed between serotype III or ST-17 GBS isolates with the rest other types.

**Table 4:** Antibiotic resistance of invasive GBS isolates in different serotypes and STs

	Serotypes					Sequence types	
	Ia (*n* = 2)	Ib (*n* = 10)	III (*n* = 53)	V (*n* = 3)	Total (*n* = 68)	ST-17 (*n* = 43)	Non-ST-17 (*n* = 25)
Penicillin	0	0	0	0	0	0	0
Ceftriaxone	0	0	0	0	0	0	0
Vancomycin	0	0	0	0	0	0	0
Linezolid	0	0	0	0	0	0	0
Ofloxacin	0	1 (10.0%)	3 (5.7%)	0	4 (5.9%)	0	4 (16.0%)^a^
Sulfamethoxazole	0	1 (10.0%)	1 (1.9%)	0	2(2.9%)	1 (2.3%)	1 (4.0%)
Tetracycline	2 (100.0%)	9 (90.0%)	51 (96.2%)	3 (100.0%)	65 (95.6%)	42 (97.7%)	23 (92.0%)
Erythromycin	1 (50%)	5 (50%)	31 (58.5%)	2 (66.7%)	39(57.4%)	26 (60.5%)	11 (44.0%)
Clindamycin	0	5 (50%)	28 (52.8%)	2 (66.7%)	35(51.5%)	25 (58.1%)	9 (36.0%)

^a^Compared to ST-17 isolates, X^2^ = 4.706, *P* = 0.030, 3/4(75.0%) ST-19 were resistant to ofloxacin

## Discussion

GBS is an important cause of neonatal sepsis and meningitis. Considerable demographic variation exists in terms of incidence and other disease burden indicators [5, 6]. The overall incidence of invasive neonatal GBS disease (0.55 per 1000 live births, 95% CI 0.44–0.69) in Guangdong between 2011 and 2014, was close to the global average estimation (0.53 per 1000 live births, 95% CI 0.44–0.62) [5]. However the incidence rate in this study is much higher than which was suggested in our previous short-term observation [10] and in other Asian countries with the incidences rate 0.14–0.41 per 1000 live births [12, 13]. Furthermore, GBS in EOD increased markedly from 2011 to 2014, reaching 0.58 per 1000 live births. The overall incidence of EOD (0.39 per 1000 live births, 95% CI 0.29–0.51) was higher than that from studies with any IAP (0.23 per 1000 live births) and lower than that from studies with no IAP (0.75 per 1000 live births) [5]. IAP was not implemented in these three hospitals during the study period. Hence, the possible reason about the incidence of LOD was more stable than EOD include: Firstly, some LOD cases might go to other hospitals rather than the studying hospitals if they moved elsewhere; doctors paid more attention to the GBS identification over time so that the blood/CSF samples could be taken more timely than before. Secondly, the clinical symptoms of LOD mostly present as neurological sequelae. Therefore, the blood and cerebrospinal fluid from patients with similar symptoms are routinely examined for the presence of bacteria in these samples. However, the symptoms of EOD are similar to respiratory infections which may be treated with broad-spectrum antibiotics before blood culture examination. After introducing the tighten regulations of antibiotic usage gradually in the study hospitals since 2012, more blood samples from potential bacteria-infected patients were examined and more GBS infection from EOD patients were identified. This may reflect the realistic situation of GBS infections especially in EOD incidences. The growing number of sterile samples were sent to the laboratory for bacterial examination using the same experimental facilities under the same examining standard in study hospitals over the studying period [14]. The increasing GBS infection rate was identified and suggested that GBS is an important pathogen of invasive neonatal infections and increases the threat to neonatal health in Guangdong in south China.

In this study the proportion of EOD (70%) is higher than that of LOD (30%). In other Asian countries such as Japan and Korea, reported that the LOD account for 75% (45/60) and 80% (125/157), respectively [13, 15]. An important risk factor for EOD is GBS colonization of maternal vaginal and rectal sites during late pregnancy. No data about GBS colonization in Guangdong has been reported. A study conducted in the other Chinese city, Beijing, showed that 7.1% of pregnant women had GBS present in the vaginal-rectal tract [16] which is lower than some developed countries (15–25%) [17, 18]. Further study to explore the relationship between EOD and maternal GBS recto-vaginal colonization will be necessary in Guangdong in the future.

The overall mortality in the present study was 7.1% (95% CI 1.1–13.2%), which is lower than the global average with 9.6% (95% CI 7.5–11.8) [5], 13.6% for EOD and 8.0% for LOD in Japan [19], 6.2% in India [20] and 20.7% for EOD and 7.2% for LOD in Korea [15]. The administration of broad spectrum antibiotics and neonatal senior nursing interventions that were provided to suspected cases of sepsis may reduce the mortality rate in the studying hospitals. In addition, the relatively low mortality might be relevant with most of cases with a normal birth weight. The proportion of LOD cases with neurological sequelae was lower than the reported 25%–35% in other countries [19, 21].

The studying hospitals provided effective anti-infection treatment for GBS cases in a timely manner and rehabilitation for cases with neurological signs, which are important to reduce the case fatality ratio and sequelae. In this study, the clinical isolated GBS strains are all susceptible to beta-lactam antibiotics including penicillin, ampicillin and cefazolin. Thus beta-lactam antibiotics are ideal to treat GBS diseases in the studying area. For the infants who are allergic to penicillin, considering high resistant rate to erythromycin and clindamycin (57.4% and 51.5%, respectively) in this study and the reported global increase in resistance to erythromycin and clindamycin over the last two decades [22, 23], other antibiotics should be chosen. Furthermore, 95.6% of isolates resistant to tetracycline and 33.8% of isolates showed multi-drug resistance to lincosamides and macrolides. The antibiotics resistance had no correlation to any particular serotypes or MLSTs which suggested the ability of antibiotic-resistance in clinical GBS strains is universal and there is no barrier of antibiotic-resistance spreading between different serotypes or MLSTs. It is necessary to unremittingly observe the antibiotic resistance of GBS in Guangdong and a guideline for reasonable usage of antibiotics against GBS is desired.

Knowledge of the serotype is crucial to the development of serotype-based vaccines against GBS disease. Serotype distribution varies by country and region [24, 25], with serotype III predominating worldwide [5, 10, 26, 27]. It is well comparable to the results of our study. In this study, the percentage of serotype III was 77.9%, among these, 81.1% were caused by ST-17, which is higher than the similar study in Japan that half (50.0%) of the serotype III strains were ST17 [28]. The use of potential GBS vaccines during pregnancy will not only overcome problems caused by IAP (e.g., no impact on LOD and antibiotic resistance), reducing the incidence and mortality of GBS disease [17, 29]. The trivalent GBS vaccine covering serotypes Ia, Ib, and III was also well tolerated and induced capsular-specific antibody responses [30], which are associated with 95.6% of GBS cases in the present study, 92.7% of GBS cases in Japan [28] and approximately 79% of GBS cases globally [5]. Furthermore, the serotyping in the present study indicated that a pentavalent conjugate vaccine covering serotypes Ia, Ib, II, III, and V could potentially prevent 100% of neonatal invasive GBS disease; these five capsular serotypes corresponded to 95% of invasive disease worldwide [5]. The findings were also consistent with a recent study among pregnant women in Beijing, which reported that serotypes III, Ia, V, and Ib accounted for 91.3% of all serotyped isolates [16].

All false-negative cases, spontaneous abortions and stillbirths caused by GBS infection might have been missed and not been included in this study. Furthermore, this study was conducted in three urban hospitals which are regarded as the best institutions for infant disease treatment in South China, it is difficult to monitor the outcomes and infections incidences after patients discharged from occasional visits. The prolonged studying period and expanded studying area will overcome the limitation of this study. Acquiring the duration and dosage of intrapartum antibiotics usage for GBS-colonized pregnant women in the course of delivery is necessary to include in the future study.

## Conclusions

This study is the first long-term epidemiology study on neonatal GBS infection in south China. Increasing GBS diseases cases and antibiotics resistance is notable in the three studying urban hospitals and serotype III and ST-17 are dominated in the GBS infection in the studying period. Our data provide the window to observe the trend of GBS infections in this area and will facilitate the development of the guidance for reasonable antibiotics usage and for the GBS vaccines in the future.

## Additional file

Additional file 1: Figure S1. Partial sequence diagram for seven house-keeping genes. adhP gene(A), pheS gene(B), atr gene(C), glnA gene(D), sdhA gene(E), glcK gene(F), tkt gene(G). (DOCX 849 kb)

## Abbreviations

CDC: Centers for Disease Control and Prevention
CI: Confidence intervals
CSF: Cerebrospinal fluid
EOD: Early onset disease
GBS: Group B Streptococcus
IAP: Intrapartum antibiotic prophylaxis
ICF: Informed consent form
MLST: Multi-locus sequence typing
ST: Sequence types
