# Supplementary figures and images for: Epidemiology of invasive group B streptococcal disease in infants from urban area of South China, 2011–2014
Source: BMC Infect Dis. 2018 Jan 8;18:14. doi: 10.1186/s12879-017-2811-0 (PMC5759214; doi:10.1186/s12879-017-2811-0)

Additional file 1: Figure S1


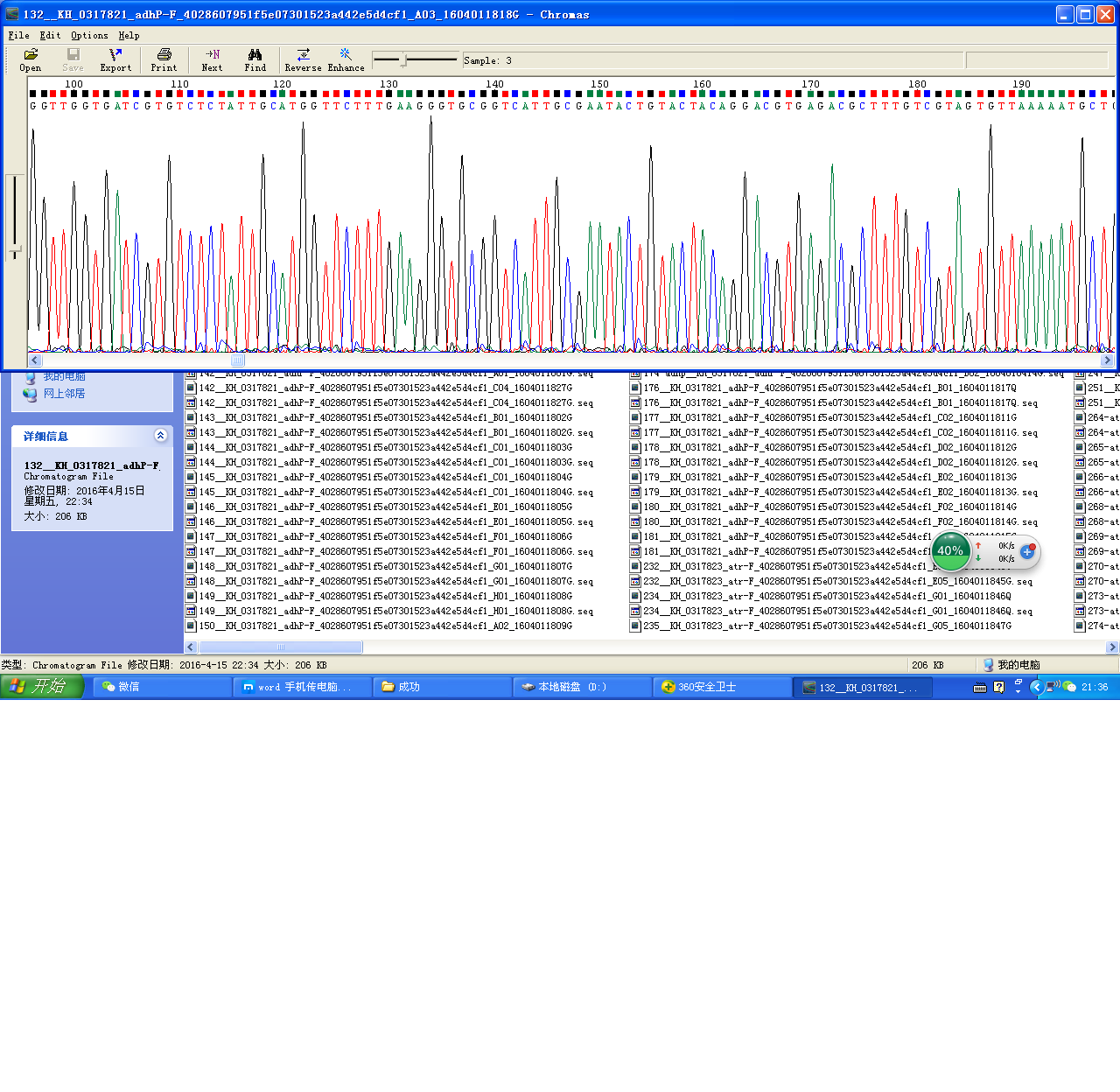

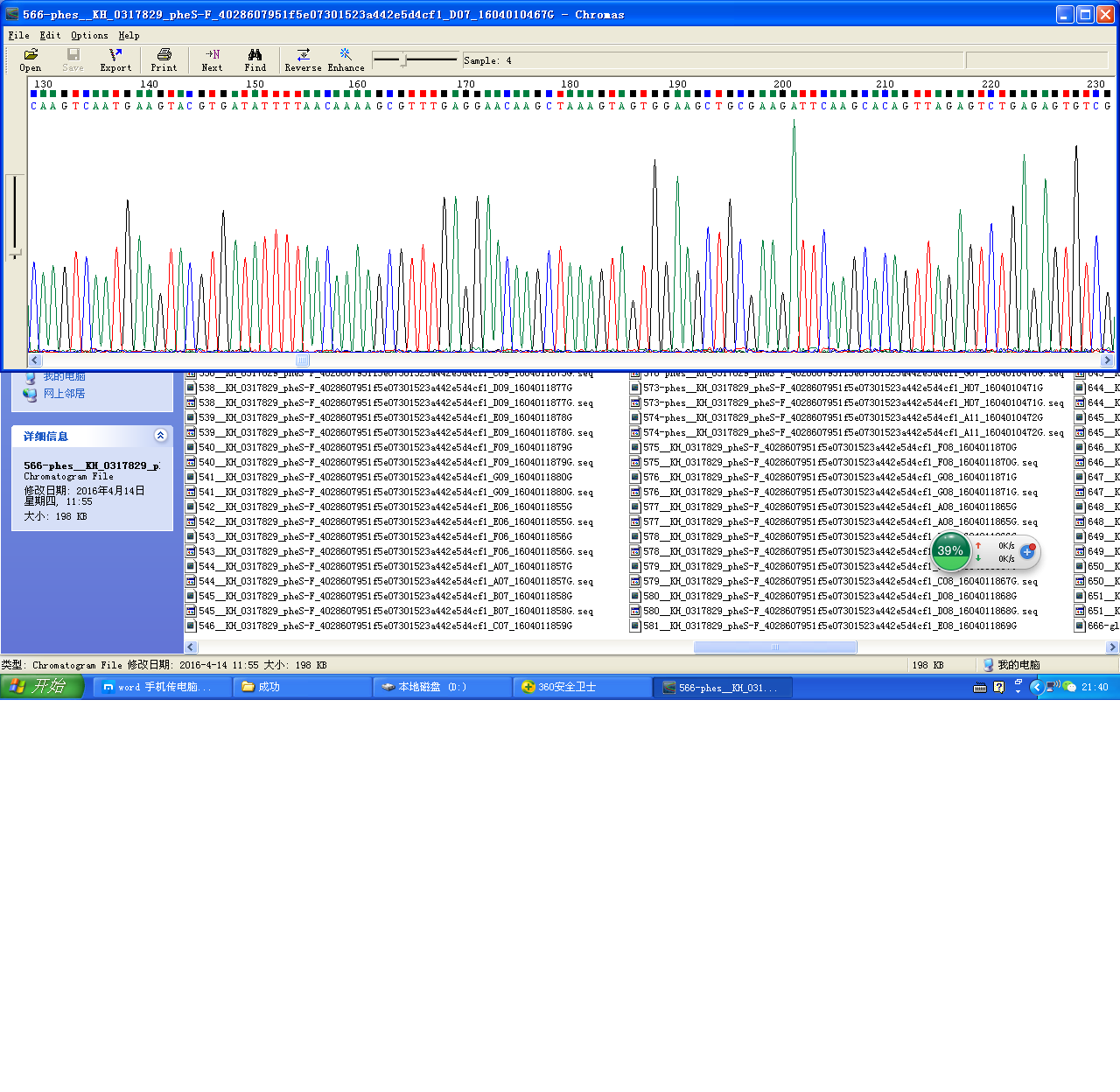


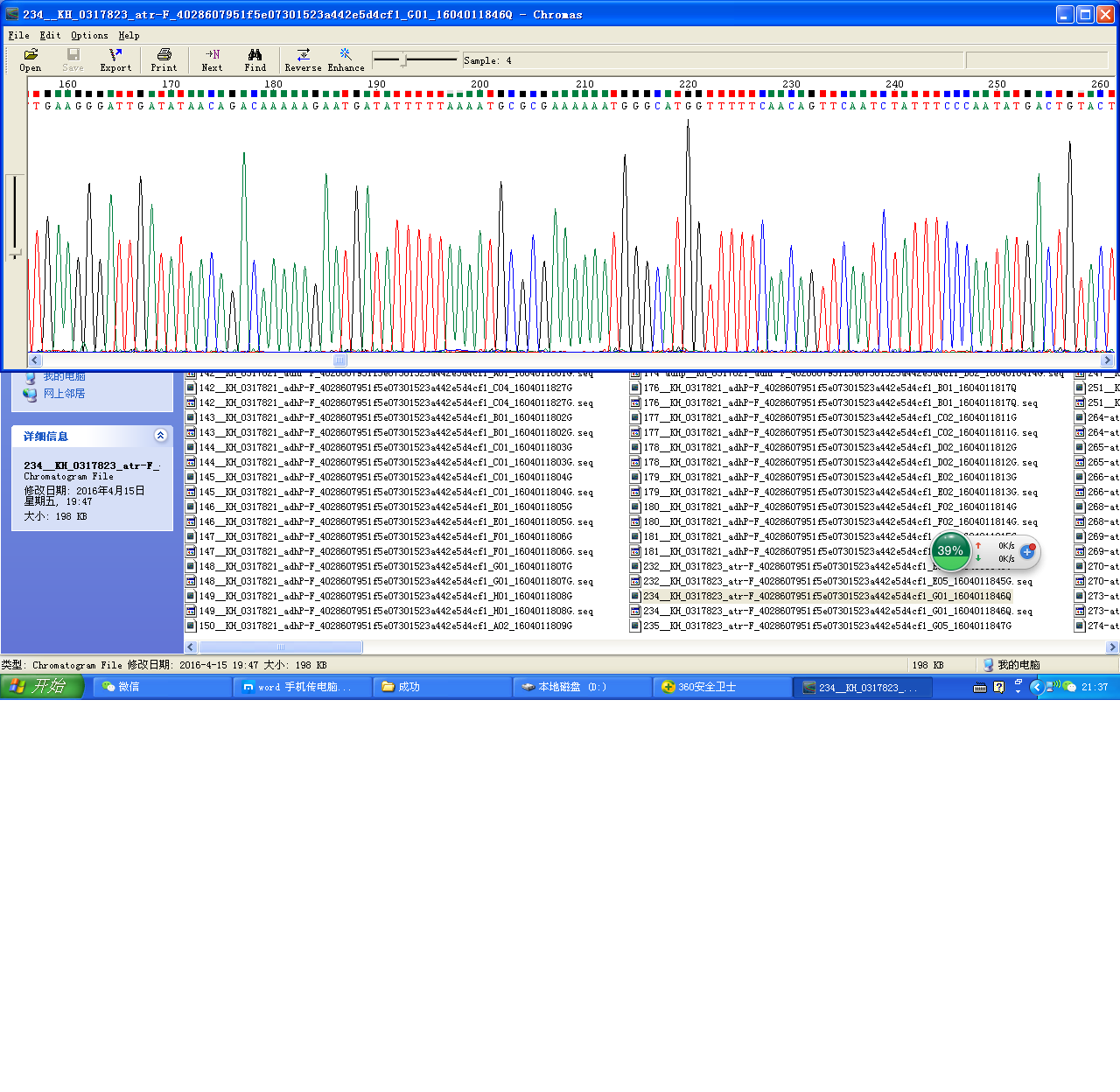

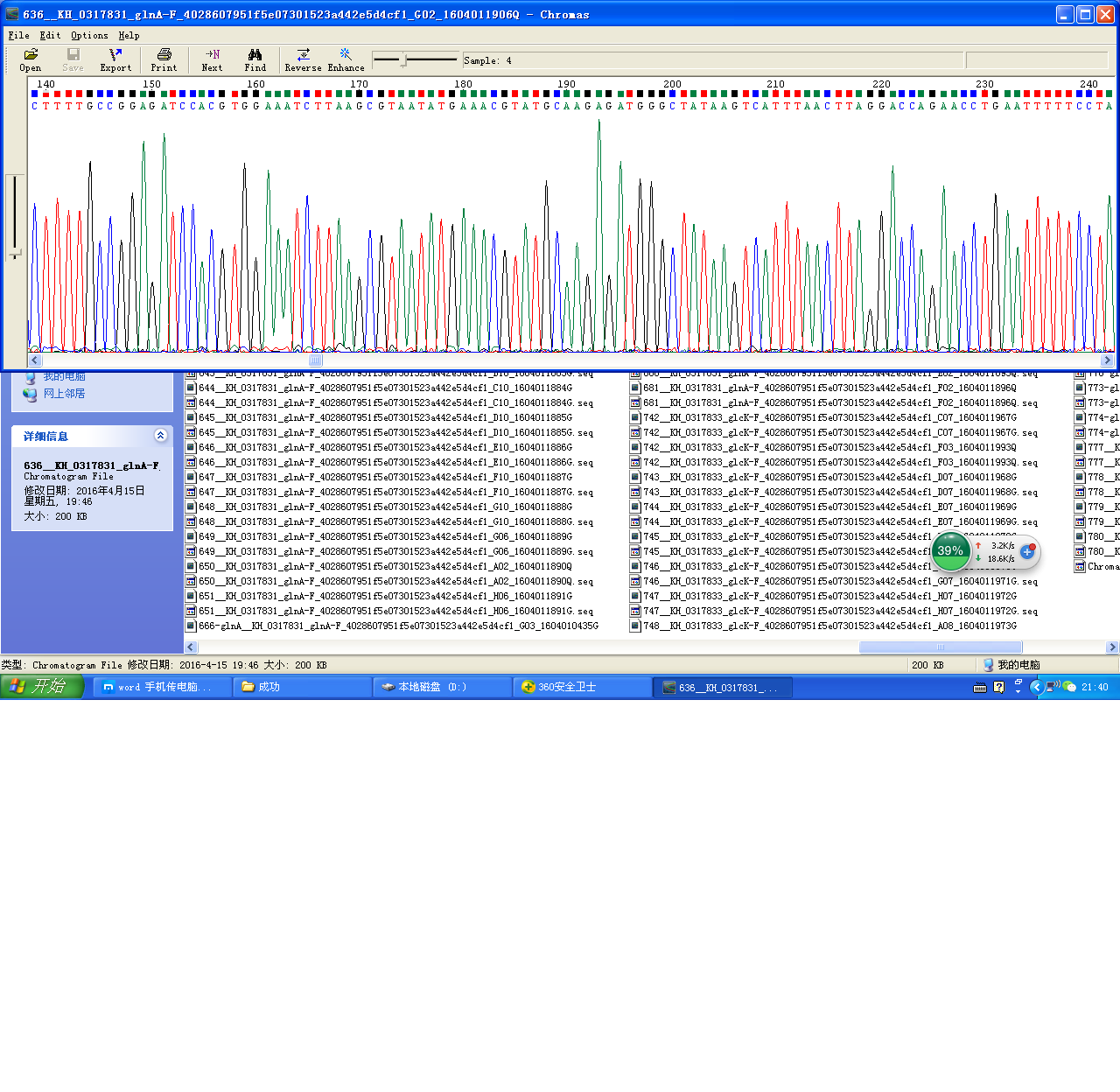


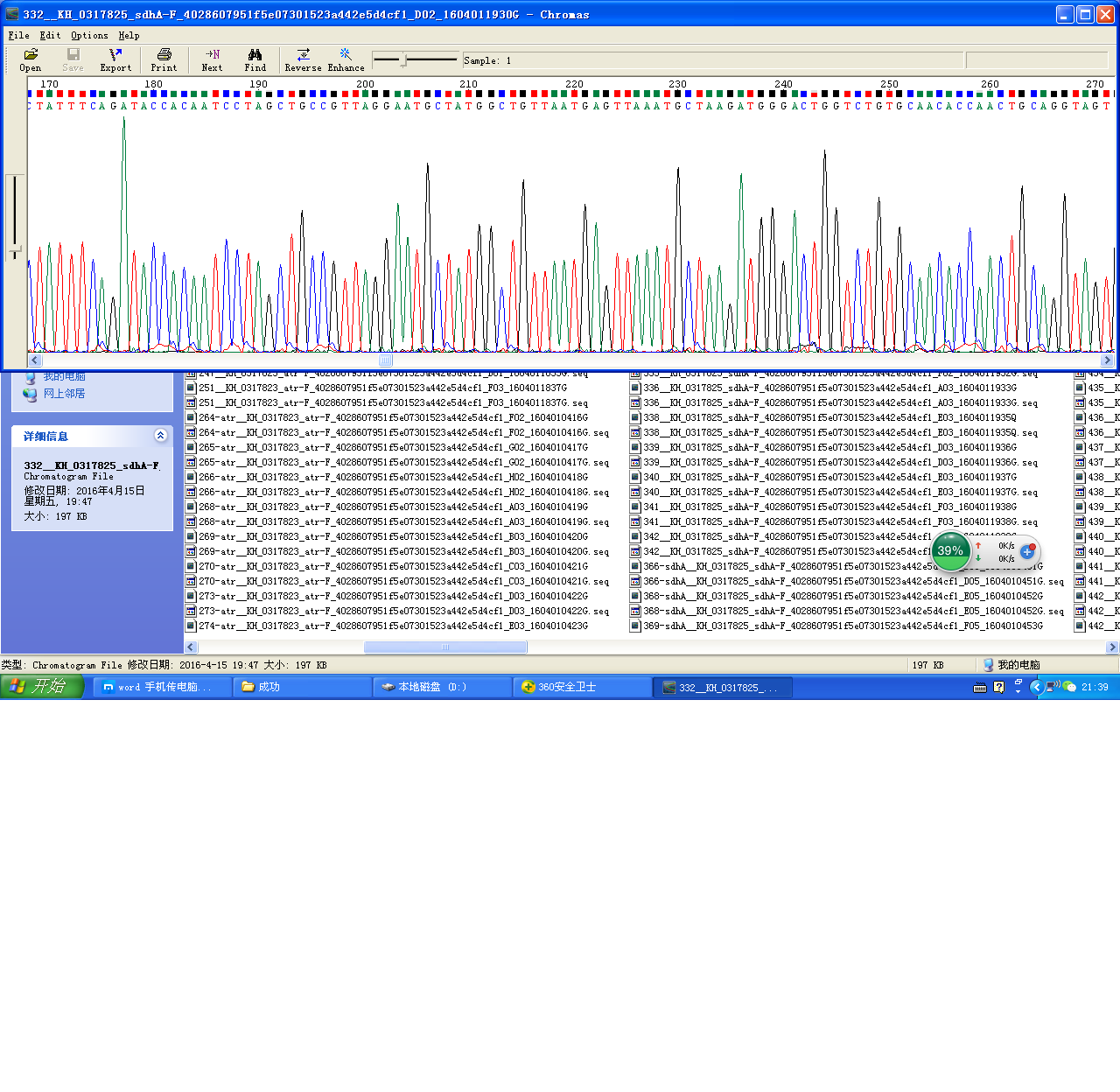

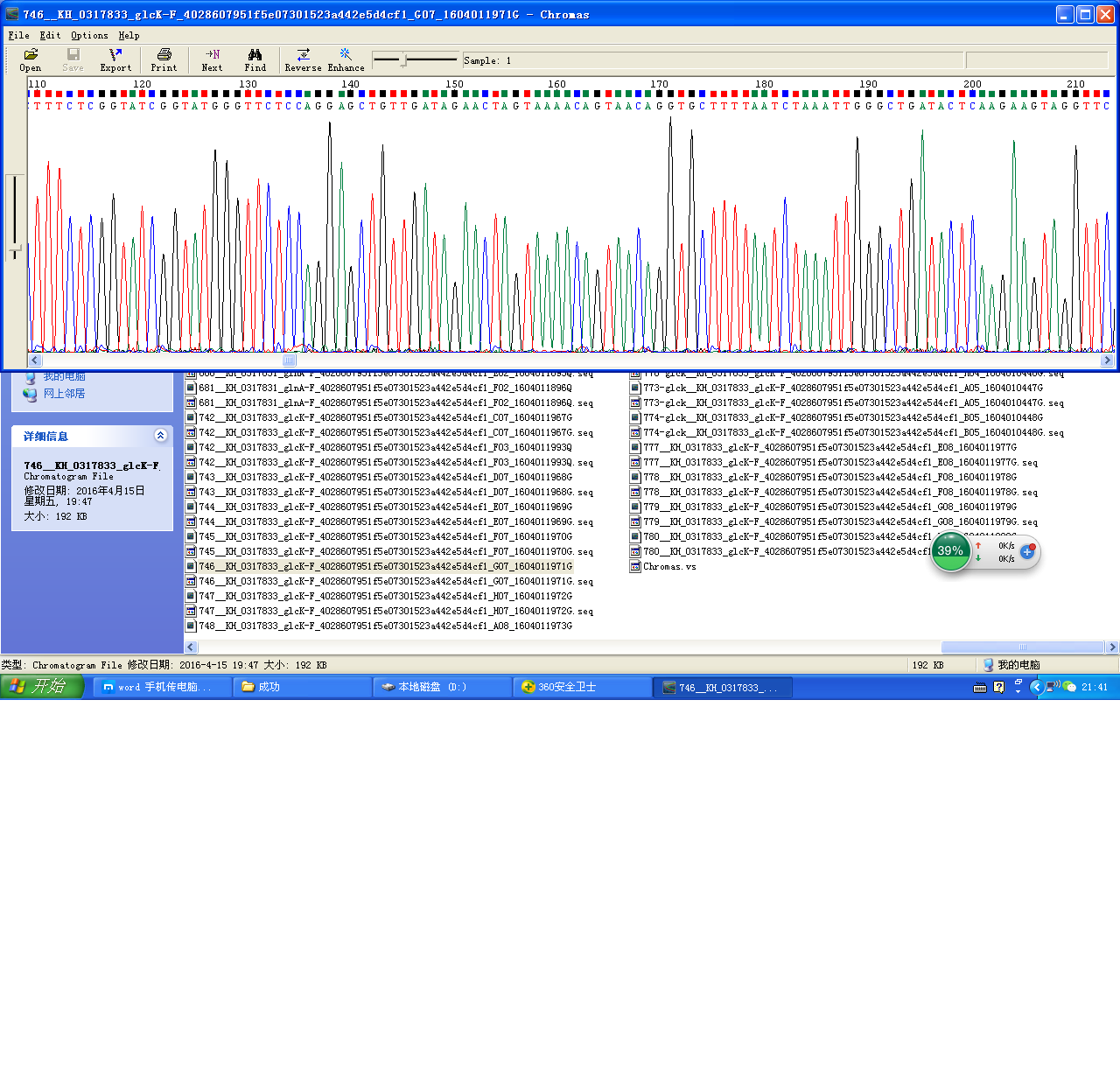


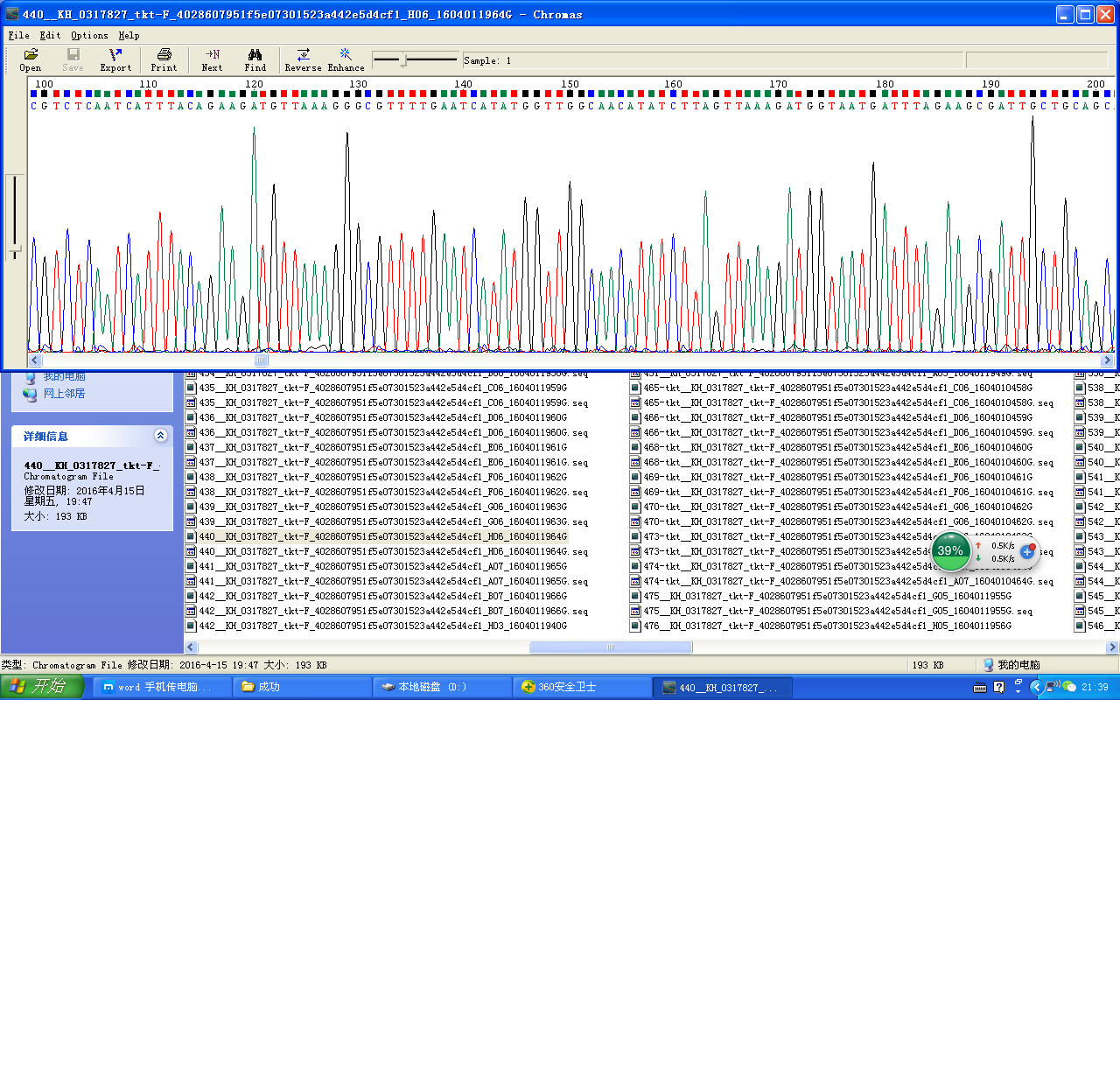

Supplement: Additional file 1: Figure S1. — Partial sequence diagram for seven house-keeping genes. adhP gene(A), pheS gene(B), atr gene(C), glnA gene(D), sdhA gene(E), glcK gene(F), tkt gene(G). (DOCX 849 kb) [file 12879_2017_2811_MOESM1_ESM.docx]
